# Thrombocytopenia after sutureless and standard stented aortic valve replacement: a retrospective analysis of risk factors, clinical course, and early outcome

**DOI:** 10.1186/s13019-024-02755-2

**Published:** 2024-04-16

**Authors:** Alicja Zientara, Mohammad Yousuf Salmasi, Bella Milan-Chhatrisha, Sharan Kapadia, Ryan Bashir, Ian Cummings, Cesare Quarto, George Asimakopoulos

**Affiliations:** 1 University Hospital Freiburg, Heart Centre, Hugstetter Strasse 55, 79106 Freiburg, Germany; 2 Department of Surgery, Imperial College London, London, UK; 3 Guy’s and St Thomas’ NHS Foundation Trust, Harefield Hospital, Hill End Road, Uxbridge, UB9 6JH UK; 4 Guy’s and St Thomas’ NHS Foundation Trust, Royal Brompton Hospital, Sydney Street, London, SW3 6NP UK; 5 Guy’s and St Thomas’ NHS Foundation Trust, St Thomas Hospital, Westminster Bridge Road, London, SE1 7EH UK

**Keywords:** Postoperative thrombocytopaenia, Aortic valve replacement, Sutureless valve replacement, Minimally invasive aortic valve replacement

## Abstract

**Objectives:**

Thrombocytopenia following Perceval aortic valve replacement has been described previously with variable outcome. Studies have lacked a robust analysis of platelet fluctuation and factors affecting it. We aimed to statistically describe the trend in thrombocyte variability as compared with conventional aortic valve replacement, and to assess predictors as well as impact on associated outcomes.

**Methods:**

One hundred consecutive patients with first-time Perceval were retrospectively compared to 219 patients after Perimount Magna Ease valve replacement. The primary outcome was the serial thrombocyte count on day 0–6. Generalized estimating equations were used to analyse the data using fixed-effect models: for the effect of the post-operative day on platelet count, and random-effect models estimating both time-variant (platelets) and time in-variant variables (valve type, age, LV function, pre-op platelet level).

**Results:**

Perceval patients were older (72 ± 1 vs 68 ± 1 years, *p* < 0.01) with higher NYHA status (3(2–3) vs 2(1–2), *p* < 0.001). Mean platelet count in the sutureless group was lowest on day 2 (91.9 ± 31.6 vs 121.7 ± 53.8 × 10^3^ µl^−1^), and lower on day 4 (97.9 ± 44) and 6 (110.6 ± 61) compared to the conventional group (157.2 ± 60 and 181.7 ± 79) but did not result in a higher number of transfusions, bleeding or longer hospital stay (*p* > 0.05). Reduced platelet count was a strong predictor of red cell transfusion in the conventional (*p* = 0.016), but not in the sutureless group (*p* = 0.457). Age (Coef -1.025, 95%CI-1.649—-0.401, *p* < 0.001) and CPB-time (Coef 0.186, 95%CI-0.371—-0.001, *p* = 0.048) were predictors for lower platelet levels.

**Conclusion:**

Considering the older patient profile treated with Perceval, postoperative thrombocytopenia does not impact on outcome in terms of transfusions, complications or hospital stay.

**Supplementary Information:**

The online version contains supplementary material available at 10.1186/s13019-024-02755-2.

## Introduction

The aetiology of thrombocytopaenia post aortic valve replacement (AVR) with the Perceval sutureless valve (Corcym Canada Corp, Burnaby, BC, Canada) has been long debated. One randomised trial and several small cohort studies comparing the Perceval with conventional stented valves have reported more profound thrombocytopenia in Perceval patients with a maximal platelet drop at either the 3rd or 4th post-operative day [1–6]. In their study of platelet response associated with various valve prostheses, Stanger et al [7] observe this trend for the Perceval compared with the Perimount Magna Ease (Edwards Lifesciences, Irvine, CA, USA), noting that only the Freedom SOLO (Sorin Biomedica, Saluggia, Italy) produced a greater decrease in platelet count than the Perceval after adjustment for confounding variables.

Similar findings of pronounced thrombocytopenia have been described in the setting of transcatheter aortic valve implantation (TAVI) [8, 9] partially associated with a higher rate of in-hospital major adverse cardiovascular events and poorer outcomes than sutureless valve implantation [6, 8, 9]. Given that the Perceval valve has a comparable stent design to transcatheter valves, it has been suggested that the origins of thrombocytopenia may be similar [3]. Explanations for thrombocytopenia reach from altered mechanical stress on platelets due to the stent to the detoxification of the Perceval leaflets with homocysteic acid (HCA) [2, 7].

Despite the reported hypotheses explaining thrombocytopaenia, clinical studies have lacked robust analysis of the longitudinal platelet trend over the post-operative course and statistically controlling for confounding variables, such as CPB time, valve size and pre-operative platelet count.

In this study, we aimed to assess the course of thrombocyte counts after surgical aortic valve replacement comparing the Perceval valve with a commonly implanted conventional prosthesis (Carpentier-Edwards Perimount Magna Ease prosthesis (PME) (Edwards Lifesciences, Irvine, CA, USA)). The aim was to investigate the clinical relevance of the thrombocytopenia in comparison with a standard biological stented prosthesis focusing on potential predictors for low platelet count and associated postoperative outcome.

## Methods

### Ethical statement

The need for ethical approval was waived by the research and ethics office at Royal Brompton and Harefield Trust, given the retrospective nature of this study.

### Study design

This was a retrospective cohort study of a prospectively collected, nationally managed database of patients undergoing AVR, with data extracted from two hospitals in the United Kingdom. All adults who received AVR ± coronary artery bypass grafting (CABG) with the Perceval or Perimount valves between 2014 and 2021 were identified. The choice between Perceval or Perimount was at the surgeons’ discretion following pre-operative assessment. As the control group patients after AVR with a PME were chosen as the standard of care.

A platelet drop between 46 and 58% of the preoperative value is reported in the recent studies with its peak reduction on day 3 or 4 [2, 3].. Post-operative platelet count in patients with sutured valves can range from 100–160 × 103 µl^−1^. Based on these parameters, we calculated that, in order to detect an even more modest reduction in platelet count (15%), a minimum sample size of 95 patients per group (sutureless vs sutured valves) would be able to detect a between-group statistical difference, with 90% power and a α statistic of 0.05. Therefore, our sample size of *n* = 100 vs 219 patients satisfy these criteria.

### Inclusion and exclusion criteria

Our institution began implanting the Perceval valve in 2014, with most operations carried out by two experienced surgeons in the department. From 2019, the new valve model, Perceval Plus, has been introduced in our department also and frequently used. Patient selection for Perceval was at the surgeons’ discretion and primarily relied on the presence of favourable anatomy for its implantation based on stenotic pathology and mainly trileaflet aortic valve anatomy. Since 2014, Perceval has been increasingly the prosthesis of choice in patients with small, calcified roots, small annulus, tricuspid or Sievers 1b and 2 valves, and in older patients. With the advent of minimally invasive procedures through smaller access, it has been increasingly implanted in our patients. Also, the patient profile of the classical Perceval patient often meets the criteria for a transcatheter option, which led despite the regular use of the prosthesis worldwide to the maintenance of a detailed process of informed consent in our unit respecting the patients’ valve choice and explaining the individual benefits.

The valve implantation was carried out as described previously [10]. The implantation of the conventional AVR was carried out with semicontinuous monofilament sutures (Prolene 2–0, Johnson & Johnson Medical N.V., Belgium 2020) or with single pledgeted sutures.

All patients undergoing first-time isolated AVR surgery were included, whilst redo cardiac surgery and patients undergoing concomitant valve or CABG operations were excluded. Furthermore, patients having Perimount size 19 and 29 mm were also excluded, due to the absence of corresponding Perceval sizes as well as patients with pure aortic regurgitation without stenotic component as their primary pathology.

### Perioperative care and (anti-)coagulation management

Patients on double-antiplatelet therapy were instructed to cease the second antiplatelet drug, mainly Clopidogrel, at least 5–7 days before the operation while Aspirin was continued throughout the procedure. Patients who were anticoagulated with Warfarin were bridged for at least 3 preoperative days with low-molecular-weight Heparin.

Peri-operative measurements by thromboelastography were carried out in specific cases of intraoperative abnormalities, i.e. prolonged bleeding, or in case of anticipated difficulties in patients with low initial platelet levels or a positive bleeding anamnesis.

Patients were managed in a post-operative recovery unit and usually spent a further day in the high dependency unit before being stepped down to the regular ward. Our standard of practice involves blood sampling immediately after patient transfer from theatre, followed by sampling on the morning of every subsequent day. As well as testing for the renal profile and electrolytes, a full blood count is also taken which includes and standardised platelet measurement.

Blood transfusions are given in the early post-operative phase based on clinical grounds. Replacement of clotting factors (fresh frozen plasma (FFP) and cryoprecipitate) and pooled platelets is guided by thrombo-elastography and by the drain output, with a preference for transfusion when mediastinal bleeding is suspected. Packed red cells are usually given when the measured haemoglobin drops below 8 g/dl. The decision for transfusion depends and is carried out on the surgical team which specifically works with the consultant and follows the departmental protocol.

### Data collection

An encrypted spreadsheet proforma was used for data collection. Demographic, pre-operative, and post-operative data for all patients, were extracted across two hospital sites. Clinical covariates and operative data were automatically extracted from the clinical database. Further operative and post-operative data were supplemented from patients’ clinical hospital records by the author RB and checked by AZ. The primary outcome measure was thrombocyte count (× 10^3^ µl^−1^). One single pre-operative thrombocyte count was used, always taken the day before surgery. Day zero represented the sample taken immediately following the patient transfer from theatre. Subsequent measurements were sampled from each patient on a daily basis between the hours of 5.00am and 8.00am: Day 1 represented the sample taken the morning following surgery and so on and so forth for each consecutive day. Secondary outcomes included blood product transfusion, overall hospital stay and incidence of complications. Since the data collection only included the perioperative stay, no further follow up was documented or analysed.

### Statistical analysis

Data distribution was analysed both qualitatively and quantitatively. Normality was assessed using the Shapiro–Wilk test. Clinical covariates were compared between the Perceval and Perimount groups using the Student t-test for parametric data, whilst the Chi-Squared test was used for non-parametric data. Data was presented as mean and standard deviations (parametric data) or medians and interquartile ranges (non-parametric data).

Since the local registry requires a complete dataset at the time of patient discharge, no missing data were detected in both groups with regards of patient characteristics and clinical outcome. The platelet count and the number of product transfusions were separately collected by the author RB and checked by author AZ using the local patient management software which at the same time is connected with the billing of the medical product.

Platelet and haematocrit (HCT) measurements were modelled in a hierarchical structure, nested into individual patient clusters and postoperative day as a progressive time factor variable. By shaping the dataset longitudinally, generalized estimating equations (GEE) were used to analyse the data using a number of models as follows:

- *Fixed-effect (FE)* model: assessing the effect of the post-operative day on platelet count was assessed
- *Random-effects (RE)* models: estimating both time-variant (platelets) and time in-variant variables (valve type, age, LV function, pre-op platelet level)

Final outcomes from regression models were provided as standardised beta coefficients with 95% confidence intervals, and a significance level set to 0.05.

## Results

In total, 100 consecutive Perceval patients and 219 patients with Edwards Perimount Magna Ease prosthesis were included in the study.

### Pre-operative covariates

Patient covariates are provided in Table 1. The Perceval group were on average older (Perceval vs Perimount: 72.4 years vs 68.2 years, *p* < 0.001), and more symptomatic (NYHA class 3 (2–3) vs 2 (1–2), *p* < 0.001). The groups were comparable for other pre-operative covariates, including gender, body mass index, diabetes, hypertension, COPD, left ventricular ejection fraction and aortic valve pathology (*p* > 0.05).

**Table 1:** Baseline patient demographic and clinical characteristics; the valve haemodynamics are shown as the predominant mechanism of the valve pathology (stenosis vs regurgitation vs mixed)

	**Perimount (%) *n* = 219**	**Perceval (%) *n* = 100**	***P value***
Age	68.2 ± 0.6	72.4 ± 0.7	< 0.001
Male	137 (62.6)	54 (54)	0.379
BMI	28.8 ± 0.4	29.3 ± 0.5	0.500
NYHA	2 (1 – 2)	3 (2 – 3)	< 0.001
Diabetes oral	31 (14.1)	14 (14)	0.442
Diabetes insulin	6 (2.7)	6 (6)	
Smoking	105 (47.9)	52 (52)	0.554
Hypertension	148 (67.6)	65 (65)	0.651
COPD	29 (13.2)	8 (8)	0.176
History of stroke	18 (8.2)	8 (8)	0.957
PVD	13 (5.9)	4 (4)	0.476
CKD	7 (3.2)	4 (4)	0.725
EF > 50%	184 (84)	88 (88)	0.379
EF 30–49%	30 (13.7)	9 (9)	
EF < 29%	5 (2.3)	3 (3)	
Valve haemodynamics (predominant pathology)			0.151
Stenosis	148 (67.6)	79 (79)	
Regurgitation	46 (21)	3 (3)	
Mixed	25 (11.4)	18 (18)	

### Operative characteristics

There was a significant difference in operative approach: 40% of the Perceval cohort had an upper mini-sternotomy, whilst only 10.5% of conventional AVR patients had this access. After matching Perceval sizing with Perimount sizing (S/21, M/23, L/25, XL/27), non-parametric testing identified no significant between group difference in the size of valve used between the two cohorts. A significantly lower operative time was identified in the sutureless group, with shorter CPB times (87 vs 93 min, *p* = 0.038) and aortic cross clamp times (58 vs 69 min), *p* < 0.001) as compared to the conventional group. Operative characteristics are summarized in Table 2.

**Table 2:** Between group comparison of operative characteristics

	**Perimount (%) *n* = 219**	**Perceval (%) *n* = 100**	***P value***
CPB time (min)	93 ± 2	87 ± 3	0.038
X clamp time (min)	69 ± 1	58 ± 3	< 0.001
Valve size			0.186
S/21	42 (19.2)	19 (19)	
M/23	87 (39.7)	31 (31)	
L/25	66 (30.1)	33 (33)	
XL/27	24 (10.9)	17 (17)	
Operative urgency			0.697
Elective	175 (79.9)	78 (78)	
Urgent	42 (19)	21 (21)	
Emergency	2 (0.9)	1 (1)	
Approach			< 0.001
Sternotomy	193 (88.1)	51 (51)	
Mini-sternotomy	23 (10.5)	40 (40)	
Right anterior thoracotomy	3 (1.4)	9 (9)	

### Post-operative platelet count and haematocrit level

**Fig. 1:**
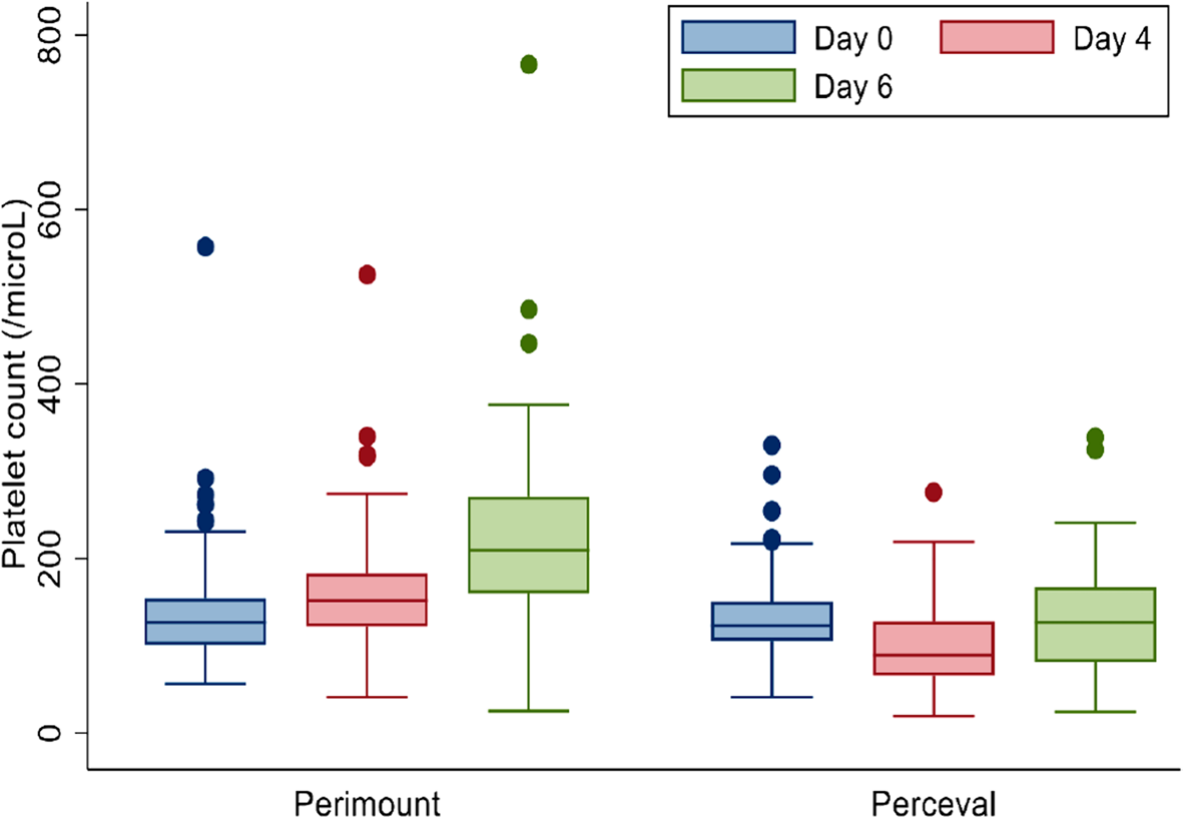
Box and whisker plot demonstrating the variation in post-operative platelet count in patients undergoing isolated aortic valve replacement with either the Perimount or Perceval valves on day 0, 4 and 6 with lower platelet count on day 4 and 6 in the Perceval group

Crude assessment of daily platelet count demonstrated on average lower values in the Perceval group when compared day by day to the Perimount cohort (Table 3, Fig. 1). The lowest average platelet count for both groups was recorded on day 2 (Perceval vs Perimount, (91.9 ± 31.6 vs 121.7 ± 53.8 × 10^3^ µl^−1^).

**Table 3:** Daily platelet count (× 10^3^ µl^−1^) and haematocrit (L/L = litre of cells per litres of blood) per valve prosthesis group, reported as means and standard deviation

**Platelet count**	**Pre-op**	**Day 0**	**Day 1**	**Day 2**	**Day 3**	**Day 4**	**Day 5**
Perceval	240.8 ± 65.2	162.1 ± 64.6	144.1 ± 54.1	91.9 ± 31.6	94.9 ± 39.5	97.9 ± 44.6	110.6 ± 60.5
Perimount	214.8 ± 75.4	135.1 ± 57.8	140.1 ± 56.2	121.7 ± 53.8	128.7 ± 62.3	157.2 ± 59.8	181.7 ± 78.6
**HCT**	**Pre-op**	**Day 0**	**Day 1**	**Day 2**	**Day 3**	**Day 4**	**Day 5**
Perceval	0.37 ± 0.06	No data	0.31 ± 0.04	0.30 ± 0.03	0.28 ± 0.03	0.29 ± 0.04	0.29 ± 0.03
Perimount	0.39 ± 0.05	0.32 ± 0.04	0.29 ± 0.04	0.28 ± 0.04	0.27 ± 0.03	0.28 ± 0.04	0.29 ± 0.04

A fixed effects model identified a significant effect of post-operative day on the platelet count for all patients (coef 7.38, 95% CI 6.44 – 8.33, *p* < 0.001). When analysing the Perimount cohort in isolation, the post-operative day also impacted platelet count (coef 11.83, 95% CI 10.72—12.93, *p* < 0.001). For the Perceval group, however, post-operative day had a statistically negative effect on the platelet count (coef -1.60, 95% CI -3.12—-0.09, *p* = 0.038).

There is a significant effect of the post-operative day on the HCT in the Perceval group (coef -0.01, 95% CI -0.01—-0.01, *p* < 0.001), which is not significant in the Perimount group (coef 0.17, 95% CI -0.08 – 0.42, *p* = 0.177).

Next, random-effects models were constructed, estimating both time-variant (platelets and HCT) and time in-variant (valve type, age, LV function, pre-op platelet level) variables (Table 4). According to this model, both older patients (coef -0.10, 95% CI -1.61—-0.38, *p* = 0.002) and the Perceval valve (coef -31.67, 95% CI -43.18—-20.16, *p* < 0.001) were predictors of reduced platelet count. In the random effects model for the HCT analysis, none of the covariates influenced the HCT level, specifically not the implantation of the Perceval valve or the post-operative day.

**Table 4:** Results of GEE model (random-effects) assessing the effect of multiple variables on platelet count and haematocrit level across 6 post-operative days

**Platelet count**	**Coef**	**Std error**	**95% CI**	***P value***
Day	7.36	0.48	6.42 – 8.31	< 0.001
Male gender	4.87	5.53	-5.98 – 15.72	0.379
Age	-0.10	0.31	-1.61—-0.38	0.002
LV function	0.06	0.27	-0.48 – 0.59	0.838
Valve choice	-31.67	5.87	-43.18—-20.16	< 0.001
**Haematocrit**	**Coef**	**Std error**	**95% CI**	***P value***
Day	0.10	0.08	-0.06 – 0.27	0.221
Male gender	0.28	0.38	-0.47– 1.03	0.462
Age	0.01	0.02	-0.03 – 0.05	0.656
LV function	0.03	0.02	-0.00 – 0.07	0.088
Valve choice	-0.32	0.40	-1.10 – 0.47	0.432

In addition, maximum likelihood models, population averaged models and between-effects models were all conducted. The latter model was used to average out the time effect between different post-operative days. In all 4 random-effects models, the effect of the Perceval valve was a consistently strong predictor of reduced platelet count over the course of the post-operative days.

### Subgroup analysis of thrombocytopaenia

Next, subgroup analysis was conducted, performing repeated random-effects models separately in the i) Perimount cohort; and ii) the Perceval cohort. For Perimount patients, CPB time and patient age had a significant negative influence on platelet count meaning that longer CPB times and older age were significant predictors of thrombocytopaenia. In contrast, for Perceval patients, neither these covariates, nor any other covariate, had a significant impact on platelet count. Valve size did not impact platelet count Perceval (coef -0.11, 95% CI -4.43 – 4.21, *p* = 0.962) or Perimount patients (*p* > 0.05).

### Post-operative complications

There was only one mortality across both cohorts, which came from the Perimount group. In-hospital stay was also similar between cohorts (median 8 days (6–10), Table 5).

**Table 5:** Between group parametric and non-parametric tests of short-term outcome

	**Perceval (%) *n* = 100**	**Perimount (%) *n* = 219**	***P value***
Return to theatres	5(5)	9 (4.1)	0.714
RBC transfused	0 (0 – 2)	0 (0 – 2)	0.617
Products^a^ transfused	0 (0 – 1)	0 (0 – 1)	0.225
In-hospital mortality	0 (0)	1 (0.5)	n.s
Postop stay (days) (IQR)	8 (6–10)	8 (6–10)	n.s

NB: ^a^1 product = one unit of platelets, FFP or RBC

Out of all patients in the study, 14 patients returned to theatre for bleeding (5/100 Perceval patients (5%) and 9/219 Perimount patients (4.1%)), demonstrating no between-group difference (*p* = 0.714). The overall products transfused was also similar between the groups, including FFP, cryroprecipitate and pooled platelets (Perceval median/IQR 0 (0 – 1), Perimount 0 (0 – 1)). Transfused packed red cells (RBC) were also similar between groups (Perceval median/IQR 0 (0 – 2), Perimount 0 (0 – 2)).

In addition, we analysed the RBC transfusion by surgical approach (minimally invasive versus sternotomy) (Table 6). Half of the patients in both groups did not require any RBC transfusion (*p* > 0.05). In the Perceval group, more transfusions were needed for sternotomy patients compared to minimally invasive approaches. The opposite effect is seen for Perimount patients, where patients operated through minimally invasive approach needed more transfusions.

**Table 6:** Subgroup analysis of Perceval and Perimount patients; RBC transfusions related to the choice of access

	**Sternotomy**	**Minimal access**	***P value***
Total number of patients	244	75	
Perceval	51	49	
Perimount	193	27	
% Patients requiring at least 1 unit RBC	123 (51%)	34 (54%)	*p* > 0.05
RBC units transfused			
Perceval	1 (0 – 3)	0 (0 – 1)	0.023
Perimount	0 (0 – 2)	1 (0 – 3)	0.031

Regression analysis (Table 7) found platelet count to be a strong predictor of RBC units transfused (*p* = 0.005). This effect persisted when analysing Perimount patients alone, although it disappeared when analysing Perceval patients. On the other hand, post-operative platelet count had no effect on return to theatre for bleeding (*p* > 0.05).

**Table 7:** Regression analysis: impact of daily platelet counts on red cell transfusion and return to theatre

	**Coef**	**Std error**	**95% CI**	***P value***
RBC transfusion (all patients)	0.0094	0.003	0.0029 – 0.016	**0.005**
RBC transfusion (Perceval)	-0.0031	0.004	-0.011 – 0.0050	0.457
RBC transfusion (Perimount)	0.012	0.0048	0.0022 – 0.021	**0.016**
Return to theatre (all patients)	1.00	0.0043	0.99 – 1.01	0.563
Return to theatre (Perceval)	0.99	0.013	0.97 – 1.02	0.624
Return to theatre (Perimount)	1.00	0.0044	0.99 – 1.01	0.457

## Discussion

This study demonstrated that implantation of the Perceval prosthesis was a consistently strong predictor of reduced platelet count over the course of the 1st to 6th post-operative day; patients with the Perceval reached the lowest platelet count on the second post-operative day. However, these factors did not translate to higher rates of clinical complications.

### Thrombocytopaenia and short-term outcomes

It may be theorised, on account of the steeper decline and slower recovery in platelet count, that the Perceval cohort would experience higher rates of post-operative bleeding than the Perimount cohort. However, the present study indicated no significant inter-cohort difference in rates of product transfusions (platelets, FFP or packed red blood cells) or re-exploration due to bleeding. Antecedent literature is conflicting with regards to incidence of these post-operative corollaries. Haeussler et al [5] and Stegmeier et al [1] demonstrate the same trends as the current study with no significant difference in blood loss, transfusion requirements, or rates of reoperation. Conversely, Albacker et al [6] report a significantly higher rate of packed red blood cell transfusion in the sutureless group and Mujtaba et al [2] describe similar findings, also reporting a significantly higher rate of platelet transfusion. This may reflect differences in clinical practice and decision making. Interestingly, we could detect a difference in the subgroup analysis between both groups and the access of choice. Perceval patients who received a full sternotomy needed more RBC transfusions while the opposite effect was seen in the Perimount group (more RBC transfusions after minimally invasive access). In general, it is important to notice that the number of RBC transfusions was very low for both groups (mean 0 with STD of 0–2) and half of the patients did not require any RBC at all.

However, the selection bias and possibly the learning curve might be two explanations: Focusing first on the Perimount minimally invasive group (more RBCs than sternotomy), the implantation of the valve by stitching is more complicated than through full sternotomy. One could assume that the classical sutured valve implantation through a small access might require a longer operation time, being technically more challenging. Secondly, patients who received a Perceval valve through full sternotomy might have a higher risk profile evaluated by the surgeon. Since the Perceval valve is the first choice for minimal access in our department, a full sternotomy in those patients might have been planned for other reasons from the beginning. Also, we did not analyse specifically the conversion rate to full sternotomy. Considering the rather lower numbers in the Perceval group, one or the other patient who has been converted from minimally invasive access to full sternotomy might have an impact on the data. In general, one needs to be careful with interpreting the data with very few numbers specifically if it comes to the RBC transfusions, which were only necessary in half of both groups and rarely exceeded one unit in the other half of the patients.

In this study, the result of the regression analysis showed that platelet count is a predictor for additional RBC transfusions only in the conventional AVR but not in the sutureless group, which may reflect the meticulous management of the Perceval patients in our department. – Indeed, the local standard of care in both included hospitals follows a clinically based concept of transfusions which is supported by the bedside measurement of the patients’ coagulation status. Postoperative decisions regarding transfusion-management are surgeon-lead and focused on daily monitoring and avoidance of unnecessary products. Clinical decisions are adjusted to the patients’ course, for instance temporary pacing wires are left in situ and cut after minimally invasive procedures rather than being pulled. Also, the removal of chest tubes is carried out early on day 1 or the latest day 2 in accordance with our local protocol documenting the lowest threshold of platelets for drain removal to be 40 × 10^3^ µl^−1^. In case the platelet count is lower, no products are given, but the patient monitored until the threshold is reached. However, being an experienced centre in Perceval implantation, the expectant management of thrombocytopaenia in Perceval patients may introduce a level of bias in our analysis. Being aware of that the thrombocytopenic effect after Perceval implantation is transient means that patients are less likely to receive blood products, which should be considered when interpreting our results.

### CPB related thrombocytopaenia

Longer CPB duration and varying levels of blood dilution following CPB have been suggested to contribute to postoperative thrombocytopenia [5, 11]. However, the present study demonstrates a significantly shorter CPB time in the Perceval group, making it unlikely to account for the diametrically opposite steeper decline in platelet count. Further, although this study noted CPB time to be a predictor for lower platelet levels, this effect did not persist in the Perceval group during sub-group analysis. In their study of the Perceval, Intuity and Sapien prostheses (both Edwards Lifesciences, Irvine, CA, USA), Haeussler et al [5] report that thrombocytopenia was observed during Sapien valve implantation despite absence of CPB, suggesting exclusive attribution to the use of CPB implausible.It is possible that Perceval-related thrombocytopenia may be partially explained by patients suffering from heparin-induced thrombocytopenia (HIT) type I and II. This argument is somewhat weakened by the fact that HIT should theoretically be distributed equally between the Perceval and Perimount groups if the anticoagulation regimens are the same. It is difficult to conclude that HIT does *not* contribute; it is often challenging or unfeasible to identify HIT and so many studies, including this one, do not account for this in their analyses [2, 7]. Since HIT may be impossible to completely exclude as a variable, it should always be considered in the clinical setting and tested.

### Valve design and thrombocytopaenia

Bioprosthetic materials may influence platelet function and number via alterations to haemodynamics or interactions at the biochemical-blood interface [7, 12]. The Perceval has a naked frame, whereas the Perimount’s frame is covered by polyester cloth [13, 14]; moreover, the Perceval’s frame is composed of nitinol (a nickel and titanium alloy), in contrast to the Perimount’s Elgiloy (cobalt superalloy) frame [7]. It is possible that direct exposure of blood to the Perceval’s metal frame leads to more significant or different effects on platelets than exposure to the polyester cloth-covered frame of the Perimount [12]. This idea is supported by Jiritano et al. and Sena et al. who compared Perceval to the cloth-covered Intuity valve [15, 16] as well as Stegmeier et al. (Perceval versus polyester-covered Labcor TLPB-A valve).

However, other naked valves, such as the Medtronic CoreValve (Minneapolis, MN, USA) [14] have either not been associated with thrombocytopenia, or have not been associated to the same extent, and the stentless Freedom SOLO often leads to worse thrombocytopenia than its stented counterparts [7, 17]. An alternative explanation is that the Perceval’s nitinol frame may release nickel and titanium ions into the bloodstream over long periods [18], but it is unclear in what concentrations, and whether this has a significant effect on platelets; for example, Pulcinelli et al. found that nickel enhances collagen-induced platelet aggregation [19], which in theory could contribute to post-operative thrombocytopenia given the tissue injury and collagen exposure seen after surgery, but Chen et al. found that nickel chloride may inhibit platelet aggregation [20].

Another explanation could be the detoxification process of the Perceval leaflets with homocysteic acid (HCA) [2], which may contribute to post-operative thrombocytopenia [7]. The bovine pericardium leaflets of the Perceval valve are fixed in a process using and later require detoxification with HCA to eliminate any residual aldehyde [2]. Short-term exposure to HCA can lead to expression of pro-apoptotic genes, progressive cellular degeneration and cell lysis in cells expressing NMDA-receptors, including thrombocytes [7]; these processes may be limited exclusively to susceptible thrombocytes following cardiopulmonary bypass (CPB) [7].

### Strengths and limitations

The main strength of this study is its robust statistical analysis using a mixed effects model in one of the largest comparative cohort studies analysing the Perceval prosthesis to date. That said, this study has limitations associated with its overall low number of patients and resultingly low event rate for bleeding/complications, thus making it difficult to draw general conclusions. Besides the focus on thrombocytopenia, the study might be likely underpowered with regards to rare clinical outcome events like stroke or bleeding complications. Mainly two surgeons are using the Perceval sutureless prosthesis of which both are also specialist in minimally invasive access and the use of one valve or the other was to their discretion demonstrating a selection bias. However, the Perceval cohort consisted of patients who were discussed previously in the heart team also considering TAVI as an option resulting in the difference that this cohort was significantly older and more symptomatic, but did not have a worse postoperative outcome compared to the conventional AVR controls. There is also the potential confounding by inter-cohort differences in baseline characteristics and the non-randomised, retrospective design. Additionally, HIT was not investigated as a potential cause of reduced platelet count. Future studies should consider investigation of pseudothrombocytopenia and assessment of platelet function.

## Conclusion

Implantation of the Perceval prosthesis is associated with a significantly larger reduction in platelet count in the immediate post-operative period than conventional stented valves, and recuperation towards pre-operative platelet levels is slower. However, clinical outcomes do not differ significantly between the valves.

### Supplementary Information

**Additional file 1.** Central Image: Platelet count with significant differences on day 4 and 6 between both valve types. *Perceval patients were significantly older.

## Data Availability

No datasets were generated or analysed during the current study.

## Abbreviations

AVR: Aortic valve replacement
CABG: Coronary artery bypass grafting
COPD: Chronic obstructive pulmonary disease
CPB: Cardiopulmonary bypass
FE: Fixed-effect
FFP: Fresh frozen plasma
GEE: Generalized estimating equations
HCA: Homocysteic acid
HCT: Haematocrit
HIT: Heparin-induced thrombocytopenia
LV: Left ventricular
NYHA: New York Heart Association
PME: Perimount Magna Ease aortic valve prosthesis
RBC: Red blood cells
RE: Random-effect
TAVI: Transcatheter aortic valve implantation
