# Supplementary material for: Thrombocytopenia after sutureless and standard stented aortic valve replacement: a retrospective analysis of risk factors, clinical course, and early outcome
Source: J Cardiothorac Surg. 2024 Apr 16;19:219. doi: 10.1186/s13019-024-02755-2 (PMC11020882; doi:10.1186/s13019-024-02755-2)

Predictors for post-AVR thrombocytopenia

for Perimount Magna:
older age & CPB time

for Perceval*
valve choice

Platelet count: Predictor for RBC transfusions in PME, not Perceval

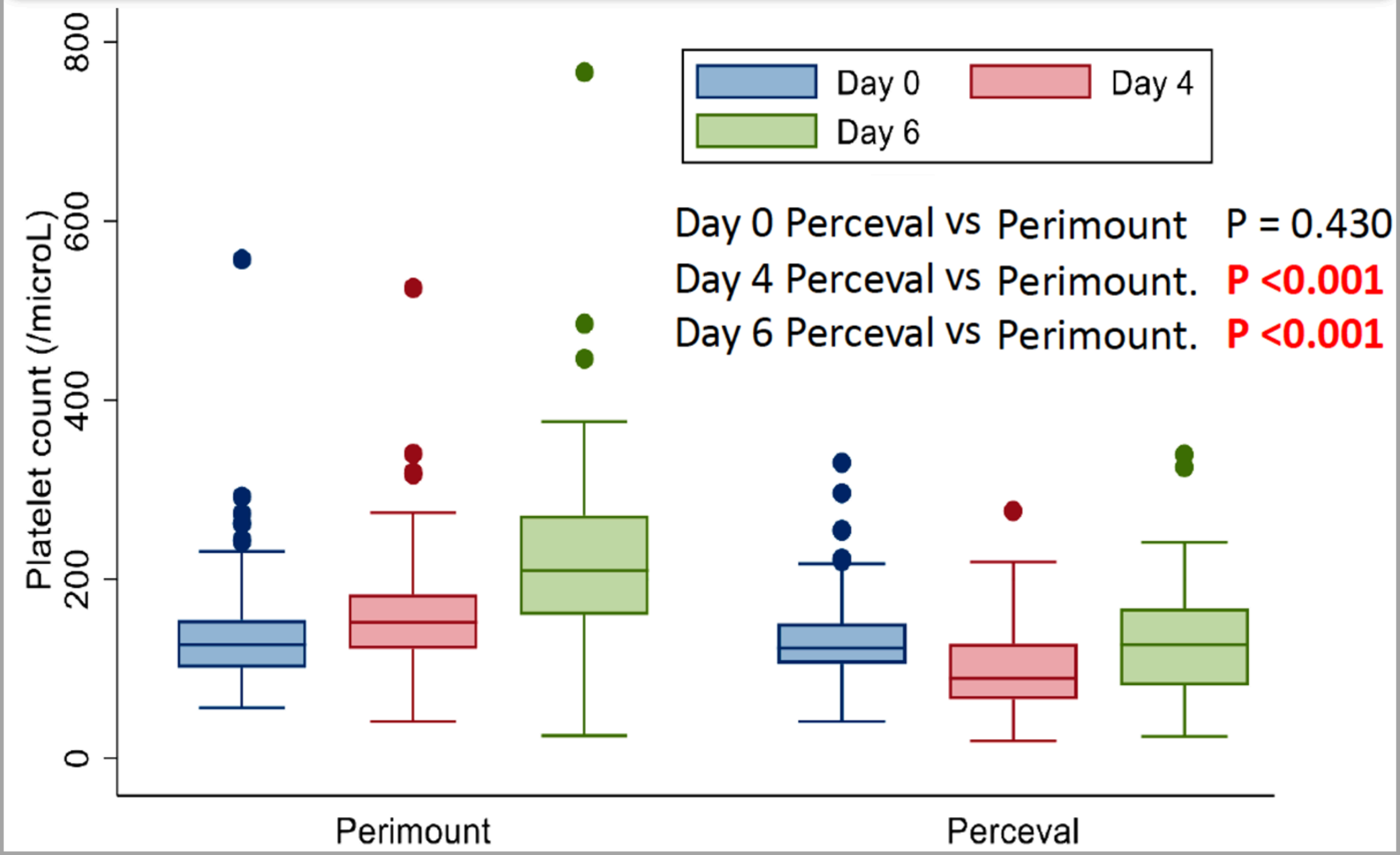

Supplement: Supplementary file 1 — Additional file 1. Central Image: Platelet count with significant differences on day 4 and 6 between both valve types. *Perceval patients were significantly older. [file 13019_2024_2755_MOESM1_ESM.pdf]
